# A New Method for Low Density Distribution Modeling and Near Threatened Species: The Study Case of *Plectrohyla Guatemalensis*

**DOI:** 10.1007/s11538-024-01315-y

**Published:** 2024-06-27

**Authors:** Miguel Ballesteros, Carlos Díaz-Avalos, Omar Hernández, Guillermo Garro

**Affiliations:** 1 Instituto de Investigaciones en Matemáticas Aplicadas y en Sistemas, Universidad Nacional Autónoma de México, Mexico City, Mexico; 2 Instituto de Biología, Universidad Nacional Autónoma de México, Mexico City, Mexico

**Keywords:** Gibbs Measures, Spatial Statistics, Markov Random Fields, Mathematical Ecology

## Abstract

We introduce a model that can be used for the description of the distribution of species when there is scarcity of data, based on our previous work (Ballesteros et al. J Math Biol 85(4):31, 2022). We address challenges in modeling species that are seldom observed in nature, for example species included in The International Union for Conservation of Nature’s Red List of Threatened Species (IUCN 2023). We introduce a general method and test it using a case study of a near threatened species of amphibians called *Plectrohyla Guatemalensis* (see IUCN 2023) in a region of the UNESCO natural reserve “Tacaná Volcano”, in the border between Mexico and Guatemala. Since threatened species are difficult to find in nature, collected data can be extremely reduced. This produces a mathematical problem in the sense that the usual modeling in terms of Markov random fields representing individuals associated to locations in a grid generates artificial clusters around the observations, which are unreasonable. We propose a different approach in which our random variables describe yearly averages of expectation values of the number of individuals instead of individuals (and they take values on a compact interval). Our approach takes advantage of intuitive insights from environmental properties: in nature individuals are attracted or repulsed by specific features (Ballesteros et al. J Math Biol 85(4):31, 2022). Drawing inspiration from quantum mechanics, we incorporate quantum Hamiltonians into classical statistical mechanics (i.e. Gibbs measures or Markov random fields). The equilibrium between spreading and attractive/repulsive forces governs the behavior of the species, expressed through a global control problem involving an energy operator.

## Introduction

Species extinction has become a major concern due to the increased rate of species loss since the past century Ceballos et al. (2015). Habitat loss and degradation is one of the most important threats to biodiversity. Amphibians are one of the most threatened groups within terrestrial vertebrates, 40.76% of the assessed species by the International Union for the Conservation of the Nature (IUCN) Red List are in a threat category (IUCN 2023), being habitat loss and modification its main threat - due to its sensitivity to environmental changes. Construction of species distribution maps as well as the screening out of factors associated to their habitat preferences and suitability are of great importance for biodiversity conservation efforts (Austin and Meyers 1996b; Jarvis and Robertson 1999; Stockwell and Peterson 2002), as those maps allow to define the areas where protection and conservation efforts are more likely to be efficient.

Distribution maps have been constructed under different approaches. These include Bayesian models (Wiens and Milne 1989; Pereira and Itami 1991; Aspinall 1992), spatial statistical methods (Wiens and Milne 1989; Pereira and Itami 1991; Aspinall 1992; Hoeting et al. 2000; Avalos 2007), ordinary generalised linear models (Austin and Meyers (1996a); Buckland and Elston (1993)), climatic envelopes (Lindenmayer et al. (1991)), genetic algorithms (Peterson et al. (1999)) and Maximum Entropy (Phillips et al. (2006)). Data records for modeling processes usually only include presence and rarely consider absence. The lack of true absence adds uncertainty to the constructed maps because the absence sites have to be inferred from the available presence records (Hoeting et al. (2000); Peterson et al. (1999); Avalos (2007)). In the case of endangered species, such uncertainty is increased due to the scarcity of geographic distribution data, and to the fact that most of the available methods rely on the assumption of spatial similarity, in which one assumes the existence of some degree of similarity between observations in neighboring areas. This is based on the principle that “Everything is related to everything else, but near things are more related than distant things” (Tobler (1970): 236).

Amphibians are the group facing the highest proportion of species in the list of threatened species IUCN (2022); Luedtke et al. (2023). In neotropical areas, anurans are a key component of the ecosystem because of their role as a node in species-interaction networks (Moritz et al. (2000)). The amphibian is a taxonomic class which is highly vulnerable to habitat fragmentation and climate change Luedtke et al. (2023). Although the World Conservation Union (IUCN) estimates that about 41% of anuran species can be classified as "endangered" or "critically endangered", for some species in these classifications there is not enough data to make a good estimation of their habitat range Urbina-Cardona and Loyola (2008). In this paper we introduce a model that can be used to construct estimations of the abundance and spatial distribution of species that are rarely observed in nature. The model is based on Gibbs measures, also known as Markov Random fields, on a grid but unlike traditional auto logistic models, we do not assume that a given pixel necessarily shows similarity with its neighbors. The approach that we introduced in Ballesteros and Garro (2022) is based on an intuitive grasp of the physical attributes of ecological phenomena, positing that individuals exhibit an inclination to be either attracted or repelled by specific environmental properties. Simultaneously, in the absence of any specific rationale for their presence in particular locations, individuals disperse uniformly across the region. Drawing inspiration from quantum mechanics, our model incorporates quantum Hamiltonians into the framework of classical statistical mechanics. The equilibrium between the dispersal and attractive (or repulsive) forces governs the behavior of the species under consideration. This equilibrium is expressed through a global control problem involving an energy operator, comprising a kinetic term (representing spreading) and a potential term (indicating attraction or repulsion). We focus on the full probability measure and implement a global control for the model, rather than examining conditional measures that contribute to a global measure. Additionally, we propose a numerical solution to address the challenges posed by Gibbs sampling (annealing), a well-known issue in situations where attaining global control becomes difficult as the number of variables increases, resulting in algorithms becoming stuck in non-optimal states.

We apply our model to the case study of *Plectrohyla Guatemalensis*, a species formerly reported as common in wet environments in the south of Mexico, Guatemala, El Salvador, and Honduras. This frog species is currently in the list of threatened species by the IUCN (2023). Recently, it has been reported a notorious decline of its population and distribution range due to habitat loss and chytridiomycosis IUCN SSC Amphibian Specialist Group (2020). *Plectrohyla Guatemalensis* is a frog species that inhabits the cloud forest from the Sierra Madre de Chiapas to the high regions in the south east of Guatemala and the mountains in the north east of El Salvador and Honduras. It has also been reported in the north of Nicaragua Faivovich et al. (2005). Its presence has only been reported at elevations ranging between 950 and 2600 meters Santos-Barrera and Canseco-Márquez (2010); Hidalgo and Ruballo-Marroquín (2012); IUCN (2022); Köhler (2011); McCranie (2017); Wilson and McCranie (2004).

**Fig. 1:**
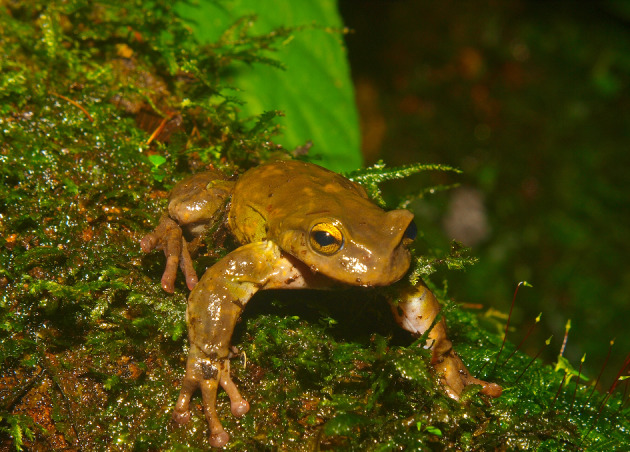
*Plectrohyla Guatemalensis* (photo by Omar Hernandez)

Plectrohyla guatemalensis inhabits cascading mountain streams in cloud forests and premountain and lower mountain forests. During the day, adults can be found in crevices near streams and arboreal bromeliads; at night, adults can be found on stream banks and rocks near streams Duellman and Campbell (1992). This species is classified as “near threatened”, according to the red list of IUCN IUCN SSC Amphibian Specialist Group (2020). This implies an expected population decline of at least $80 \%$ within the next years due to fragmentation, habitat loss, hybridization, competition with introduced species, pollution, parasites, and specially chytridiomycosis produced by the fungus *batrachochytrium dendrobatidis* (see Mendelson et al. (2004); Mendoza-Almeralla et al. (2015); Muñoz Alonso (2010); Santos-Barrera (2004); Urbina-Cardona and Loyola (2008)). The population of this species declined significantly in Mexico, Guatemala and El Salvador, and it is more abundant in Honduras (see Mendoza-Almeralla et al. (2015); Santos-Barrera (2004)). Efforts have been made to pin point the current distribution range of *Plectrohyla Guatemalensis* and to screen out factors related to it.

The rest of the paper is structured as follows: In Sect. 2 we describe the study area where the field work was carried out, the methodology used for the sampling in the field work and we present the collected data. In Sects. 3 and 4 we outline the mathematical model based on Gibbs measures that we use to estimate spatial distribution and abundance of the species *Plectrohyla Guatemalensis*. In Sect. 5 we present our results and, finally, Sect. 6 is devoted to the conclusions.

## Study Area

**Fig. 2:**
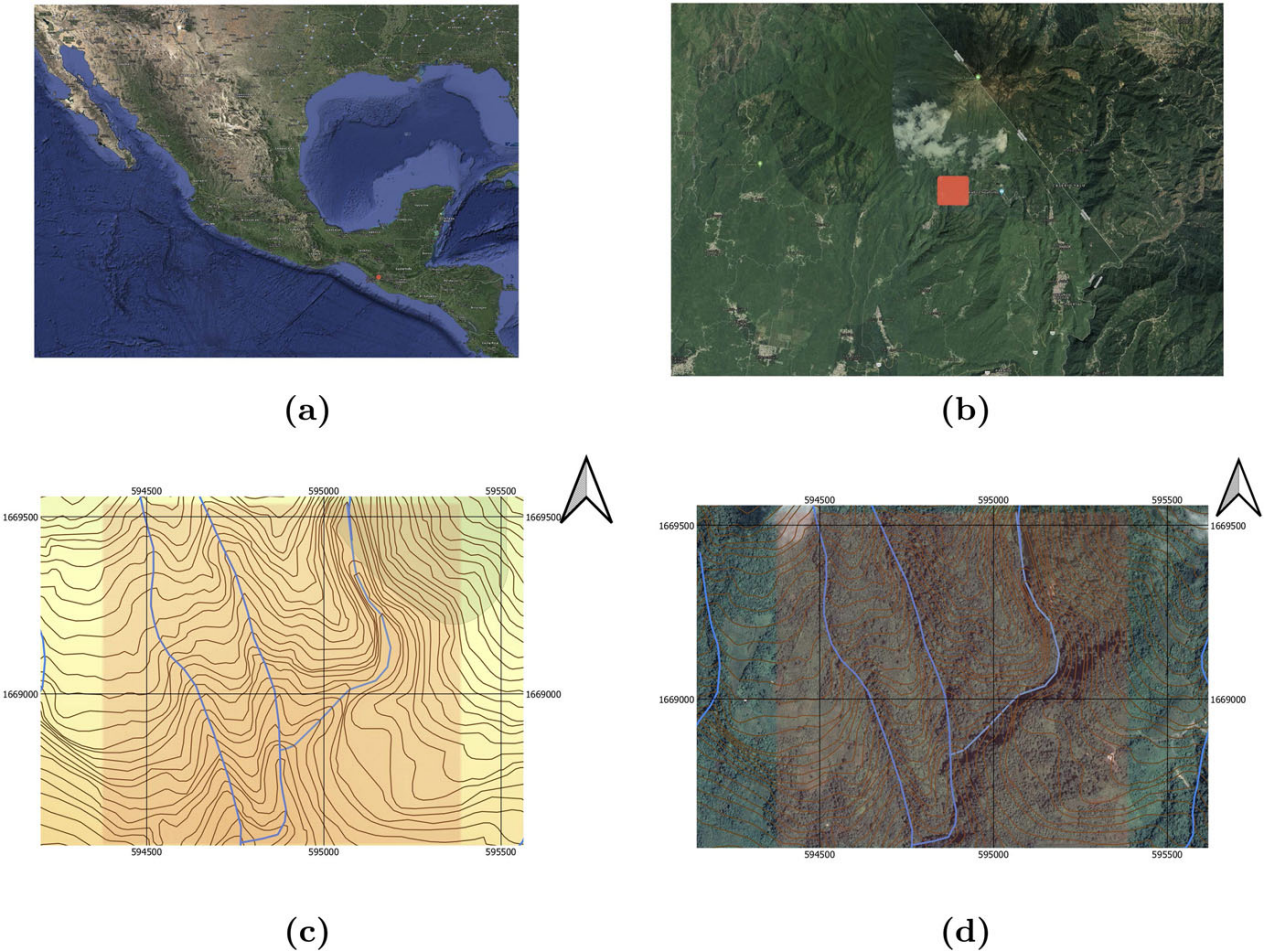
Location of the study area in Mexico as a red dot **a** and close up of the study area **b** near the border of Mexico with Guatemala. Figure **c** shows contour lines (brown) and the streams (blue). Figure **d** shows a satellite image from Google Maps in which we extracted the piece of the map corresponding to the study region (Google Maps. (2023). Tacana Volcano. Retrieved from https://www.google.com/maps/@15.1129024,-92.1386786,22624m/data=!3m1!1e3?entry=ttu)

The study area is located in an area close to the Tacaná Volcano, near Chiquihuites town, Chiapas, Mexico (see Fig. 2). It is a rectangular area, inside the polygon defined by the diagonal vertices $x=(594378,1668578)$ and $y=(595383,1669533)$ UTM coordinates in the WGS 84 15N projection. The study area location is shown in Fig. 2 (see Section 2.1.1 in Ballesteros and Garro (2022) for more details). The study area is crossed by a river that bifurcates into two branches. Details on the field work can be found in Aguilar (2019), which consisted of 10 field trips during the year 2018, where a very small portion of the study region was sampled (here we provide simulated samplings for the full region). We partition the study area in a rectangular grid with square cells of 5*m* side length.

### Data Collection

We use data from Aguilar (2019), where 10 field trips during the year 2018 were carried out on February 2nd, February 8th, March 1st, March 8th, June 6th, July 5th, August 19th, August 26th, November 7th and December 18th, 2018. The dates were chosen to cover up possible seasonal changes in the spatial variation of the target species.

A fixed 75*m* transect along the river bed and 5 square parcels outside the river bed, with side length of 5*m*, were surveyed, and the number of individuals of *Plectrohyla Guatemalensis* were recorded during every field trip together with their location. The total number of individuals registered on the river bed was 17, and the corresponding number for the parcels outside the river bed was 3 individuals. The coordinates of the parcels are presented in Table 1.

**Table 1:** Coordinates of the parcels

Parcels	UTM Coord	Grid Coord
1	(595008.5, 1668868.5)	(133, 126)
2	(595048.5, 1668903.5)	(126, 134)
3	(595068.5, 1668948.5)	(117, 138)
4	(595113.5, 1668973.5)	(112, 147)
5	(595163.5, 1668998.5)	(107, 157)

## Model Based on Gibbs Measures for *Plectrohyla Guatemalensis*

**Fig. 3:**
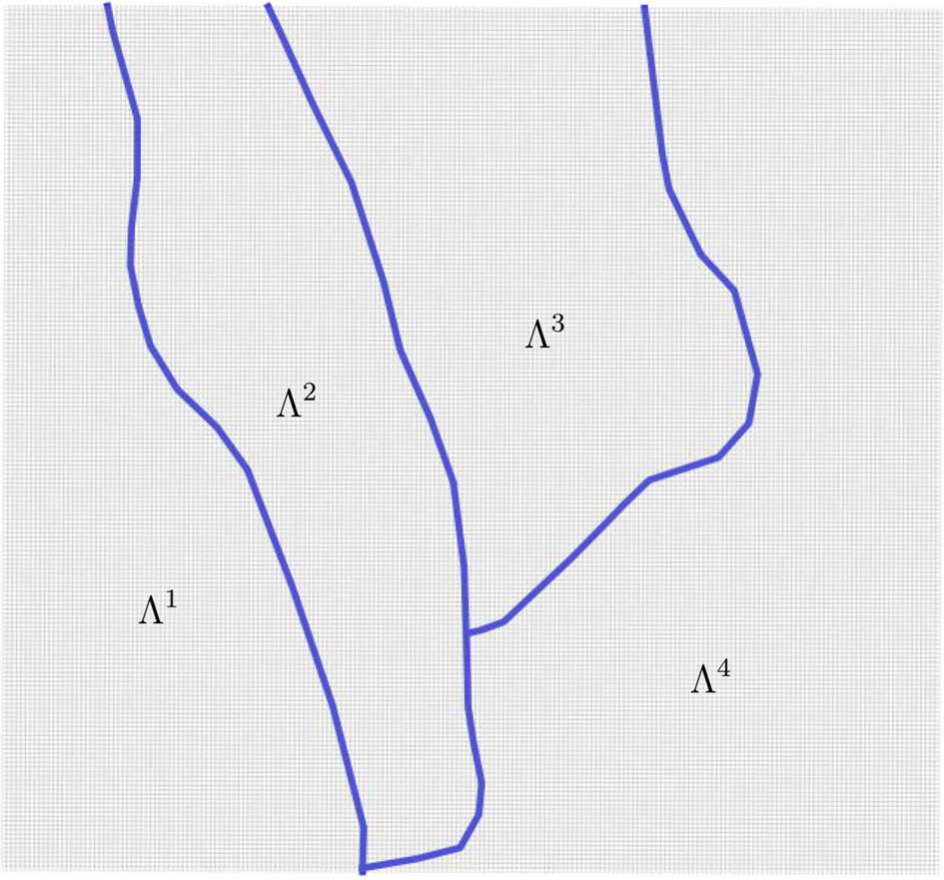
The River grid *R* (blue) and the Regions $\Lambda ^4$, $\Lambda ^3$, $\Lambda ^2$, $\Lambda ^1$

Our goal is to obtain a distribution map for the species of interest in the region depicted in Fig. 2c, which we denote by $\mathcal {D}$. We apply the method that we introduced in Ballesteros and Garro (2022) with important new features (see Hoeting et al. (2000); Peterson et al. (1999); Avalos (2007) for other approaches). In short, the region $\mathcal {D}$ is a rectangular area with sides of length 960m and 1010m. We construct a grid (that we denote by $\Lambda$) on $\mathcal {D}$ with $192 \times 202$ cells (each one of size $5m\times 5m$ - see Sect. 4 ). The grid is crossed by a river, which produces a partition in four connected components that we denote by $\Lambda ^1, \, \Lambda ^2, \, \Lambda ^3, \, \Lambda ^4$ (see Fig. 2c, Sect. 4, and Fig. 3 ). We denote the cells covering the river by *R* (see Sect. 4) . Then, the full grid is the disjoint union of $\Lambda ^1,..., \Lambda ^4$ and *R*. Since $\Lambda ^1,..., \Lambda ^4$ are disconnected from each other, we can study them separately (see Remark 4.5 and Ballesteros and Garro (2022)).

Let *K* be the yearly average per cell density on the river, calculated as follows: In Aguilar (2019) it is reported that 75 meters were sampled on the river, and the width of the river is approximately 5 meters. Then, we consider that this piece of the river contains 15 cells. The year-average density per cell is just the total number of individuals collected during 2018 on the river divided by 15 (the total number of sampled cells on the river) times 10 (the number of dates when field work was carried out). We denote this number by

3.1 $$\begin{aligned} K := \text {year-average density of individuals per cell on the river } =\, 0.113. \end{aligned}$$

In this manuscript we consider *K* to be an adimensional quantity (a numerical value). However, although we are not modeling a phenomenon in physics but a phenomenon in biology, we might still want to associate a notion of “physical units”. The appropriate units for *K* would be *individuals* / *(cells* $\times$ *number of fieldwork trips )*.

Our method aims to reconstruct the distribution from sampled data. It consists in the following two steps:

- Step 1. Reconstruction of the distribution on the river grid from sampling data (75 ms along the river ).
- Step 2. Reconstruction of the distribution outside the river using Markov random fields (Gibbs measures) with boundary conditions on the river given by Step 1.

Reconstructing river data is crucial, because it provides boundary values for the Markov random fields. Our approach is unique because we are not associating to each cell an integer number corresponding to the number of individuals, which is the usual procedure, as we did in Ballesteros and Garro (2022). Instead, we associate real numbers belonging to the interval

$$\begin{aligned}{}[0, K], \end{aligned}$$

corresponding to year-average densities (here we assume that the densities attain their maximal value on the river cells, which is reasonable because the species that we study are attracted to water bodies). This is an important input which is used for the first time in this paper. It is necessary because there are very few observations (we recall that the species that we study is endangered). On each field trip we might find only 2 individuals, and from this it is impossible to reconstruct any distribution. Our approach considers 10 fieldwork trips (the whole year) at once. Nevertheless, our observations are still too seldom (see (3.1)) and our reconstruction procedures have to take this into consideration. The first part of our reconstruction procedure (Step 1) cannot use a Poisson process, as we did in Ballesteros and Garro (2022). The reason is that Poisson processes produce sparse occurrences. These occurrences generate artificial clusters around them on Step 2, once we introduce Markov random fields in order to reconstruct the distribution outside the river (this a consequence of neighbor interactions in the Gibbs measures). As we mention above, the solution that we address is to drop the idea of associating individuals to the cells (positive integer numbers) and to use instead densities (positive real numbers in the interval [0, *K*]). Then, we simply assume that river is homogeneous (the density along the river is constant) and we do not require Poisson processes (as we do in Ballesteros and Garro (2022)). The 75 ms sampled on the river corresponds to a small portion of it. However, we assume that this portion represents the full river and take the quantity $K = 0.113$ to be the year-average density of individuals on all river cells. This accomplishes Step 1 of our reconstruction method, which is mathematically very simple but conceptually important and innovative. Step 2 of our reconstruction procedure is quite involved. Specifically, the Hamiltonian generating the Gibbs measure is the sum of a kinetic term (promoting spreading) and a potential energy (attractive force to the river whose strength is parametrized by a coupling constant *g*). This functional is denoted by *H* and it is introduced in (4.6). We take conditional probabilities in order to fix the river data of Step 1. This generates a new Gibbs measure for which Regions 1, 2, 3, and 4 are decoupled (see Sect. 4.4). We focus on Region 4 where sampling data is available. We study the low temperature regime in which the probability is concentrated on the minimal values of the Hamiltonian (this realizes the equilibrium between spreading and attractive forces, which is the main idea of our method). The coupling constant *g* is chosen in order to match collected data outside the river (this is a new feature with respect to Ballesteros and Garro (2022)). The mathematical details, reconstruction procedures and results related to Step 2 are presented in the forthcoming sections. Our method (step 1 and Step 2) produces values for the year-average densities for all cells of the study region, which is our main goal (see Sect. 5.3 and Fig. 6). Our results permit us to simulate random realizations modeling observations of individuals resembling two-dimensional Poisson processes (see Sect. 4.7.1 and Figs. 7, 8).

The original idea of using Markov random fields in geo-statistics was introduced in Besag (1972, 1974), and it plays a prominent role in the field. However, a global control using Hamiltonians inspired in quantum mechanics is a new feature of our method. For near threatened species, observations are rare and, therefore, the collected data is reduced to a few individuals. Other spatial statistical methods such as geostatistics or spatial point process do not perform well with sparse data Armstrong (1998); Besag (1972, 1974); Chilès and Delfiner (2012); Cressie (2015); Geman and Geman (1993); Isaaks and Srivastava (1989); Matheron (1962); Wackernagel (2013); Waller and Gotway (2004), and new insights to our method in Ballesteros and Garro (2022) must be introduced. The problem is that from only local properties of Markov random fields (as in Besag (1972, 1974); Grimmett and Welsh (1990); Cressie and Lele (1992); Dobruschin (1968); Kaiser and Cressie (2000); Kindermann and Snell (1980); Li (2009); Sherman (1973); Spitzer (1971); Zhu et al. (2005)), the most that we can get is clusters around the few observations, unless we introduce covariate data in the model as in Avalos (2007). These clusters are unreasonable for low density species because species density is so low that chances of finding clusters of individuals are extremely low. As we described above, we address and solve this problem in the present manuscript for the study case that we analyze.

## Mathematical Framework

### Grid, States and Neighbor-Structure

In this section we present the mathematical model that was already introduced in Ballesteros and Garro (2022). For the convenience of the reader and for the sake of completeness, we state here in full detail our mathematical formalism.

The grid, $\Lambda$, introduced in Sect. 3 is given by

$$\begin{aligned} \Lambda : =\Big \{ 1,\cdots , M \Big \} \times \Big \{ 1,\cdots ,N \Big \}, \end{aligned}$$

where $$M=192$$ and $$N=202$$.

We define (see (3.1)):

4.1 $$\begin{aligned} \Gamma := [0,K] , \hspace{1cm} \Omega := \Gamma ^{\Lambda }. \end{aligned}$$

Every element $$ \omega \in \Omega $$,

4.2 $$\begin{aligned} \omega : \Lambda \rightarrow \Gamma , \end{aligned}$$

is named a state (or a configuration). We set

$$\begin{aligned} \omega _{i,j}\equiv \omega ((i,j)), \end{aligned}$$

which is the year-average density of individuals on the cell (*i*, *j*).

We use the symbol

$$\begin{aligned} N(i,j):=\Lambda \cap \{ (i, j-1),(i+1, j), (i, j+1), (i-1, j)\}, \end{aligned}$$

to denote the (first order) neighbors of the cell (*i*, *j*). Moreover,

$$\begin{aligned} (l,m)\sim (i,j) \end{aligned}$$

signifies that (*i*, *j*) and (*l*, *m*) are neighbors ($( l, m ) \in N(i,j))$.

The set of cliques is

4.3 $$\begin{aligned} \mathcal {C}&: = \Big \{ \{(r, s+1) , (r, s)\} \Big | (r, s+1) , (r, s) \in \Lambda \Big \} \nonumber \\&\quad \cup \Big \{ \{(i +1, j), (i, j) \} \Big | (i +1, j), (i, j) \in \Lambda \Big \} . \end{aligned}$$

It follows that

$$\begin{aligned} N(l,m)=\{(i,j)\in \Lambda :\{(l,m),(i,j)\}\in \mathcal C\}. \end{aligned}$$

### Energy Functionals (Hamiltonians)

#### Free Energy (Free Hamiltonian)

We denote by

4.4 $$\begin{aligned} H_{0}(\omega ):= \sum _{\{ i, j \} \in \mathcal {C}} (w_i - w_j)^2,\qquad \omega \in \Omega , \end{aligned}$$

the free Hamiltonian (recall (4.3)).

#### The Potential Well (Potential Energy)

The potential well that we consider is given by

4.5 $$\begin{aligned} V_g(\omega ): = g \sum _{ j\in \Lambda } d_{j}^2 \omega _j, \qquad \omega \in \Omega . \end{aligned}$$

In (4.5), $d_j$ is the distance of the cell *j* to the river, i.e.

$$\begin{aligned} d_j=M_j+A_{j}. \end{aligned}$$

$$M_j$$ is the horizontal distance from the cell *j* to the river and $$ A_{j}$$ is the vertical distance from the cell *j* to the point of the river that minimizes the horizontal distance. Inspired in harmonic oscillator in quantum mechanics, we choose the square in (4.5). However, this is clearly not the only choice. The quadratic behavior is taken as a hypothesis and we leave only one free parameter to be adjusted (or estimated) from fieldwork data (namely *g*). The reason why we leave only one free parameter is that sampling data outside the river is very limited and it does not allow an accurate description of the the potential well. As it is customary in physics in many situations, we assume that it is quadratic (i.e, we take only the fist term in a Taylor series centered at the minimizer).

#### Full Energy (Full Hamiltonian)

The full Hamiltonian is given by

4.6 $$\begin{aligned} H(\omega ):= H_{0}(\omega ) + V_{g}(\omega ),\qquad \forall \omega \in \Omega . \end{aligned}$$

### Markov Random Fields (Probability Measure - Gibbs Measure)

The probability measure on $\Omega$ is given by

4.7 $$\begin{aligned} \frac{1}{Z} \exp \left\{ -\frac{1}{T}H(\omega )\right\} =: \pi _{T, g}(\omega ) \equiv \pi (\omega ),\qquad \forall \omega \in \Omega , \end{aligned}$$

see Sect. 4.2, where

$$\begin{aligned} \sum _{\omega \in \Omega }\exp \left\{ -\frac{1}{T} H(\omega ) \right\} =: Z \end{aligned}$$

is the partition constant.

#### Markov Random Fields

Given an element $\iota \subset \Lambda$, and for every $q \in \iota$, we set $W_q: \Gamma ^{\iota } \rightarrow \mathbb {R}$ by

4.8 $$\begin{aligned} W_{q}(\omega ) = \omega _q. \end{aligned}$$

Moreover, for each $\upsilon \subset \iota$, we define

4.9 $$\begin{aligned} W_{\upsilon } := \{W_{q} : q \in \upsilon \}. \end{aligned}$$

The random variables $W_q$ constitute a Markov random field, representing the year-average density of individuals.

### River Data (the Attractor): Region Hamiltonians ($H^i$, $i \in \{1,2,3,4 \}$)

#### River Grid and Regions

The river grid, $R \subset \Lambda$, is the set of cells touching the river, see Fig. 3. *R* divides $\Lambda$ in four connected components: $\Lambda ^1$, $\Lambda ^2$, $\Lambda ^3$ and $\Lambda ^4$ (see Fig. 3). They are called Regions 1, 2, 3 and 4. It follows that

$$\begin{aligned} R \cup \bigcup _{i=1}^{4}\Lambda ^{i} = \Lambda . \end{aligned}$$

We set

$$\begin{aligned}{} & {} \Lambda \setminus R =: \Lambda ^R,\\{} & {} \Gamma ^{\Lambda ^R } =: \Omega ^R, \hspace{1cm} \Gamma ^{ \Lambda ^{i} } =: \Omega ^i, \hspace{1cm} \forall i \in \{1,2,3,4 \}. \end{aligned}$$

We define $\varvec{\omega }^{R}: R \rightarrow \Gamma$ by (see (3.1))

$$\begin{aligned} \varvec{\omega }^{R} (\ell ):= 0.113 = K. \end{aligned}$$

$\varvec{\omega }^{R}$ represents the year average density of individuals on the river cells. This is an important difference from our previous work Ballesteros and Garro (2022), where the distribution of the river cells is given by Poisson simulations and the river cells take values on the number of individuals—a finite set of integer numbers—instead of year-average densities, which belong to the interval [0, *K*].

#### Boundary Conditions on the River

For every $\omega$ in $\Omega ^R$, we set $\omega ^R$ by

4.10 $$\begin{aligned} \omega ^R_{q} : = {\left\{ \begin{array}{ll} \omega _{q}, &{} q \in \Lambda ^R \\ \varvec{\omega }^{R}_{q}, &{} q \in R. \end{array}\right. } \end{aligned}$$

We define

4.11 $$\begin{aligned} \pi ^R(\omega):=\mathbb {P}_{\pi }\Big ( W_{ \Lambda ^R} = \omega \mid W_{ R} = \varvec{\omega }^{ R}\Big ) =\frac{\pi ( \omega ^R)}{\displaystyle \sum _{b \in \Omega ^R}\pi ( b^R)}. \end{aligned}$$

##### Definition 4.1

(Hamiltonian with Boundary Conditions on *R*) We set, for $\omega \in \Omega ^R$,

4.12 $$\begin{aligned} H_0^R(\omega ):&=\sum _{\begin{array}{c} t_1\sim t_2 \end{array}}(\omega _{t_1}-\omega _{t_2})^2 +\sum _{\begin{array}{c} t\sim s\\ s\in R \end{array}}(\omega _t-\varvec{\omega }^R_s)^2,\end{aligned}$$

4.13 $$\begin{aligned} V_g^R(\omega )&=g \sum _{ t \in \Lambda ^R} d_t^2 \omega _t. \end{aligned}$$

The Hamiltonian with boundary conditions on the river is given by

4.14 $$\begin{aligned} H_0^{R}(\omega )+ V_g^R(\omega )=: H^R(\omega ). \end{aligned}$$

##### Remark 4.2

A simple calculation leads us to

4.15 $$\begin{aligned} \pi ^{R}(\omega ) = \frac{1}{Z^{R}} e^{-\frac{1}{T} H^{R}(\omega ) }, \end{aligned}$$

where

$$\begin{aligned} \sum _{\omega \in \Omega ^R }\exp \left\{ -\frac{1}{T}H^R(\omega )\right\} =: Z^R. \end{aligned}$$

#### Region Hamiltonians ($H^i$, $i \in \{1,2,3,4 \}$)

For every $\omega$ in $\Omega ^i$, we define

4.16 $$\begin{aligned} \pi ^i(\omega):=\mathbb {P}_{\pi }( W_{ \Lambda ^i} = \omega \mid W_{ R} = \varvec{\omega }^{ R}). \end{aligned}$$

##### Definition 4.3

(Hamiltonians $H^i$, $i \in \{1,2,3,4 \}$) For every $\omega \in \Omega ^i$, we set

4.17 $$\begin{aligned} H_0^i(\omega ):&=\sum _{\begin{array}{c} t_1\sim t_2 \end{array}}(\omega _{t_1}-\omega _{t_2})^2 +\sum _{\begin{array}{c} t\sim s\\ s\in R \end{array}}(\omega _t-\varvec{\omega }^R_s)^2,\end{aligned}$$

4.18 $$\begin{aligned} V_g^i(\omega )&=g \sum _{ t \in \Lambda ^i} d_t^2 \omega _t, \end{aligned}$$

and

4.19 $$\begin{aligned} H_0^{i}(\omega )+ V_g^i(\omega ) =:H^i(\omega ). \end{aligned}$$

The free Hamiltonian $$ H_0^{i} $$ is minimized in the constant state, i.e. it promotes spreading. The potential-well energy $$V_{g}^{i} $$ is minimized when all individuals are on the river. Minimizing $$H^i$$ produces an equilibrium between having all individuals homogeneously distributed allover the region and having all individuals on the river. The coupling function *g* describes the strength of the attraction force to the river (we estimate it with experimental data).

##### Definition 4.4

For every $\omega \in \Omega ^R$ (or $\omega \in \Omega ^i$) and $\iota \subset \Lambda ^R$ (or $\iota \subset \Lambda ^i$ ), we use the symbol

$$\begin{aligned} \omega |_{\iota } \end{aligned}$$

to denote the restriction of $\omega$ to $\iota .$

##### Remark 4.5

After a direct calculation, we obtain

4.20 $$\begin{aligned} \pi ^{i}(\omega ) = \frac{1}{Z^{i}} e^{-\frac{1}{T} H^{i}(\omega ) }, \end{aligned}$$

where

$$\begin{aligned} \sum _{\omega \in \Omega ^i}\exp \left\{ -\frac{1}{T}H^i(\omega )\right\} =: Z^i. \end{aligned}$$

A simple calculation leads us to

4.21 $$\begin{aligned} \sum _{i} H^i(\omega |_{\Lambda ^i})= H^R(\omega ) , \hspace{2cm} \forall \omega \in \Omega ^R, \end{aligned}$$

and

4.22 $$\begin{aligned} \prod _{i = 1}^4 \pi ^{i}(\omega |_{\Lambda ^i})=\pi ^{R}(\omega ) . \end{aligned}$$

The above equations imply that the random variable $W_{\Lambda ^i}$ is independent of $W_{\Lambda ^j}$, whenever $i \ne j$. Consequently, we can study separately the regions $\Lambda ^4, \Lambda ^3, \Lambda ^2, \Lambda ^1$, i.e. they are decoupled.

The parameter *T* is the temperature. When *T* is small, the most probable states are concentrated among the less energetic states. However, it is a hard problem to get access to such low energy states (this is a minimization problem). We use different values of *T* in order to optimize this parameter with the help of Gibbs sampling (see Sect. 5.1). As we specify in Definition 4.3, low energy states are the ones that model the equilibrium between spreading and being attracted to the river. In Ballesteros and Garro (2022), we justify our procedure. More precisely, we show that after an appropriate selection of the parameter *T*, the Gibbs sampling procedure leads us to an (approximate) minimizer in the free case (g=0) which is a state that is essentially constant. We slowly increase the parameter *g* and observe that the outcomes of the Gibbs sampling simulations slowly get concentrated in the river to the point that when *g* is large enough we obtain states that are supported in a very small vicinity of it. This is exactly what we need for modeling, because we realize our intuitive idea that the states (minimizers) that we obtain represent an equilibrium between spreading and being attracted to the river and that we can manipulate the coupling constant in order to get what we expect. This also shows that we reach a global control of the measures that we use.

### Cells Ordering and Gibbs Sampling

For every $i \in \{1,2,3,4 \}$, we define a sequence $(\ell ^i_{n})_{n \in \mathbb {N}}$, with $\ell ^i_n \in \Lambda ^i$ and with the following properties.

- $(\ell ^i_{n})_{n \in \mathbb {N}}$ is periodic, with period equal to the total number of cells in $\Lambda ^i$, that we denote by $m^i$. Moreover, $\ell ^i_1, \dots , \ell ^i_{m^i}$ are all different and they cover the full Region $\Lambda ^i$.
- The first elements of $(\ell ^i_{n})_{n \in \mathbb {N}}$ cover all neighbors of the river, the first and the second elements cover all neighbors of the first elements. The first, second and third elements cover all neighbors of the first and the second elements. We proceed in the same manner until we cover all cells in $\Lambda ^i$.

The previous properties do not precisely specify a sequence, but they are the important qualities of our sampling scheme. In Section 4.5.1 of Ballesteros and Garro (2022) we specify the sequence that we use, for region $\Lambda ^4$ (the others are similarly constructed). We sample the Gibbs measures that we use with the help of the well-known Markov chain Monte Carlo (MCMC) algorithm called Gibbs sampling (this method was introduced in Geman and Geman (1993)). Although general definitions of Gibbs sampling are available in the literature, in the following definition we make precise the Gibbs sampling algorithm that we use in this manuscript.

#### Definition 4.6

(Gibbs Sampling) A Gibbs sampling (for the problem that we study) is a sequence of states $(\omega ^{i,n})_{n \in \mathbb {N}\cup \{ 0\}}$ in $\Omega ^i$ defined by the following: We choose an initial state $\omega ^{i,0}$. If $\omega ^{i,0}, \dots , \omega ^{i,n-1}$ are defined, we randomly take $x \in [0,K]$ and set $\tilde{\omega }$ to be the state that coincides with $\omega ^{i, n-1}$ in all cells with the exception of $\ell _n$, where it takes the value *x*. We choose $\omega ^{i, n} = \tilde{\omega }$ if $H^i( \tilde{\omega }) < H^i( \omega ^{i, n-1} )$. Otherwise, we randomly select $y \in (0,1)$. If $y \le e^{ -\frac{1}{T} ( H^i(\tilde{\omega }) -H^i( \omega ^{i, n-1}) ) }$, then $\omega ^{i, n} = \tilde{\omega }$. If this is not the case, $\omega ^{i, n} = \omega ^{i, n-1}$.

The importance of Gibbs sampling is that $(\omega ^{i, n})_{n \in \mathbb {N}\cup \{ 0 \} }$ can be used to calculate expectation values of the number of individuals on each cell:

#### Theorem 4.7

(Ergodic Theorem - Theorem C Geman and Geman (1993)) Suppose that we replace [0, *K*] by a finite set $\Gamma$. Suppose that $(\theta ^n)_{n \in \mathbb {N}\cup \{ 0\}}$ is a Gibbs sampling in $\Lambda ^{i}$. Then

4.23 $$\begin{aligned} \text {Expected value of the density of individuals on}\,p = \lim _{n \rightarrow \infty } \frac{1}{n} \sum _{m = 1}^{n} \theta _{p}^{m} , \end{aligned}$$

almost surely.

The ergodic theorem is also addressed in Robert and Casella (2013) and Winkler (2012). In these sources, it is not proved in the way we need it because here we have [0, *K*] instead of a finite set $\Gamma$. However, in practical situations, the ergodicity is generally useless because convergence rates are too slow most of the times (see Theorem 1 in Hajek (1988) and Theorem 5.1.4 in Winkler (2012)). It is not the intention of the present manuscript to apply such a theorem but to find a method that represents the idea of an equilibrium between attraction and spreading, in such a way that the attraction forces can be precisely controlled in order to match experimental data. This is exactly what we obtain in Sect. 5, and this is the core of our method introduced in Ballesteros and Garro (2022): We globally control our measures, in such a way that for the free case we do approach the energy minimum and as the coupling constant increases we continuously move from a homogeneous situation to the point where all individuals are on the river. This suggests that we are already in the regime where the ergodic theorem (and annealing) is valid, but the proof of this is not useful in this manuscript because we achieve numerically what we want in terms of modeling (the ergodic theorem, and annealing serve as inspiration).

### Parameter Estimation

#### Auxiliary Temperatures: $T_1$ and a Range of $T_2$

As we explain above, our model relies on using low energy states of Hamiltonians in order to estimate the probability distribution of individuals. The minimizing technique that we utilize is finding the most probable states for the measures $\pi ^i_T$, using Gibbs sampling in the low temperature regime. Here we focus on Region $\Lambda ^4$ because field work was carried out in this region. As we explain in Ballesteros and Garro (2022), in the case that $g=0$ (which we call the free case) the minimizer of the energy functional is the constant state. We use this case for calibration because we have an explicit expression for the state we want to reach through Gibbs sampling. In Ballesteros and Garro (2022), we explain in full detail this procedure. We make different choices of the temperature *T* and consider corresponding Gibbs sampling (finite) sequences with $1000000 \times m_4$ iterations, where $m_4 = 13715$ is the number of cells in Region $\Lambda ^ 4$, and initial state equal to zero. We select the temperature $T_1$ that minimizes (numerically) the average energies of the last $10000\times m_4$ iterations.

Averaging these last iterations, we obtain a new state, that we call $\varvec{\omega }_0'$, whose energy is already close to the minimum. We take this as an initial state and descend the temperature starting from $\varvec{T}_1$ in order to get a more accurate estimate for the energy—the lowest that we can achieve numerically–and this defines a new temperature $\varvec{T}_2$. This describes a discrete temperature descent procedure (or annealing) that can be iterated in order to get better estimations, however in our case $\varvec{T}_2$ gives already good results.

#### Estimating of g and T

We denote by ${\hat{ \varvec{g}}}$ and ${\hat{ \varvec{T}}}$ the best estimates that we obtain for the parameters *g* and *T*. *g* is the most important parameter in this manuscript, because it fixes the attraction strength to the river and in order to determine it we use data collected in the parcels, see Table 1.

We denote by $P \subset \Lambda ^4$ the set of parcels. Since all parcels belong to Region 4, we use this region to fix the coupling constant *g*. We temporarily make explicit the dependence on *g* of the Gibbs sampling sequences.

4.24 $$\begin{aligned} (\omega ^{i,n})_{n \in \mathbb {N}\cup \{ 0\}} \equiv (\omega ^{i,n; g})_{n \in \mathbb {N}\cup \{ 0\}}. \end{aligned}$$

We start with Gibbs sampling simulations with temperature $T= T_1$ and $g = 0$ and slowly increase *g*. We estimate the expectation value of the year-average number of individuals on the parcels *P* with the averages of the last

$$\begin{aligned} {\mathcal {M}}:= 10000\times m_4 \end{aligned}$$

iterations of Gibbs sampling simulations of $1000000\times m_4$ iterations (and initial state zero):

4.25 $$\begin{aligned} \text {Expected number of individuals on}\,P = \int _{P} \omega d \pi ^4(\omega ) \approx \frac{1}{{\mathcal {M}}} {\sum _{n=990001\times m_4}^{1000000\times m_4}} \sum _{p \in P} \omega ^{i,n; g}_p. \end{aligned}$$

**Fig. 4:**
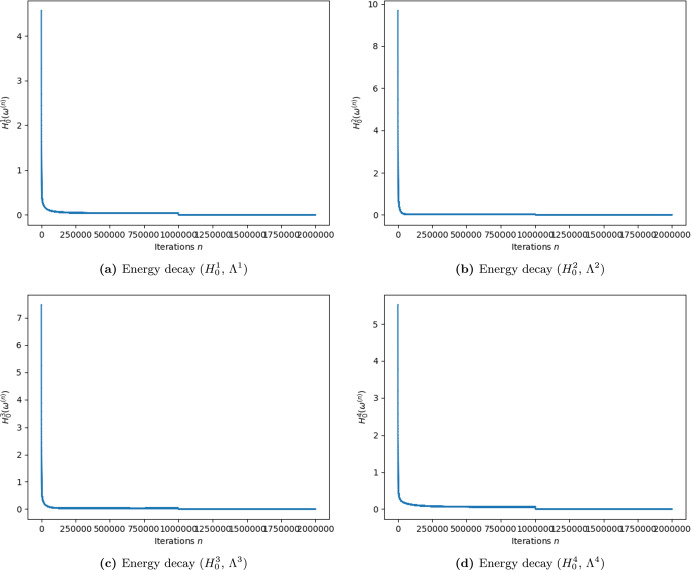
Free energy with temperature ${\hat{ \varvec{T}}}$, as described in Section 4.6, for 2000000 Gibbs sampling iterations of the form $k=m_ik$, for $k=1,...,2000000$ and $m_i$ is the number of cells of each Region $\Lambda ^i$, $i=1,2,3,4$

**Fig. 5:**
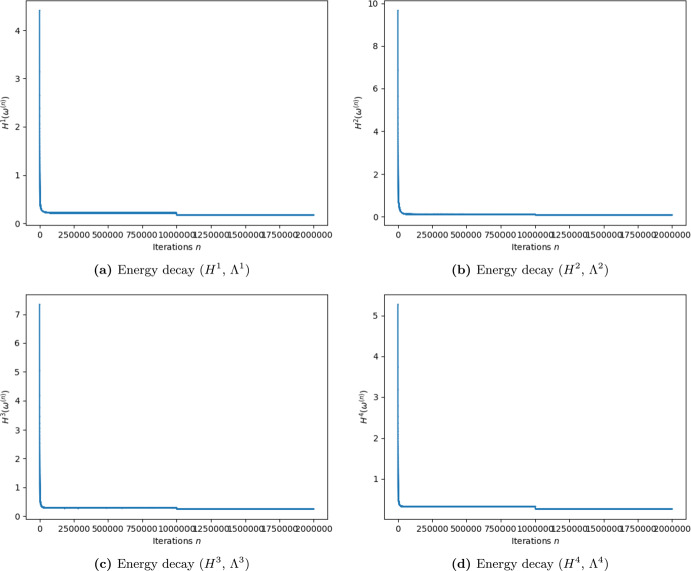
Energy Stabilization in the Perturbed Case: Energy with temperature ${\hat{ \varvec{T}}}$ and coupling constant ${\hat{ \varvec{g}}}$, as described in Section 4.6.2, for 2000000 Gibbs sampling iterations of the form $k=m_ik$, for $k=1,...,2000000$ and $m_i$ is the number of cells of each Region $\Lambda ^i$, $i=1,2,3,4$

**Fig. 6:**
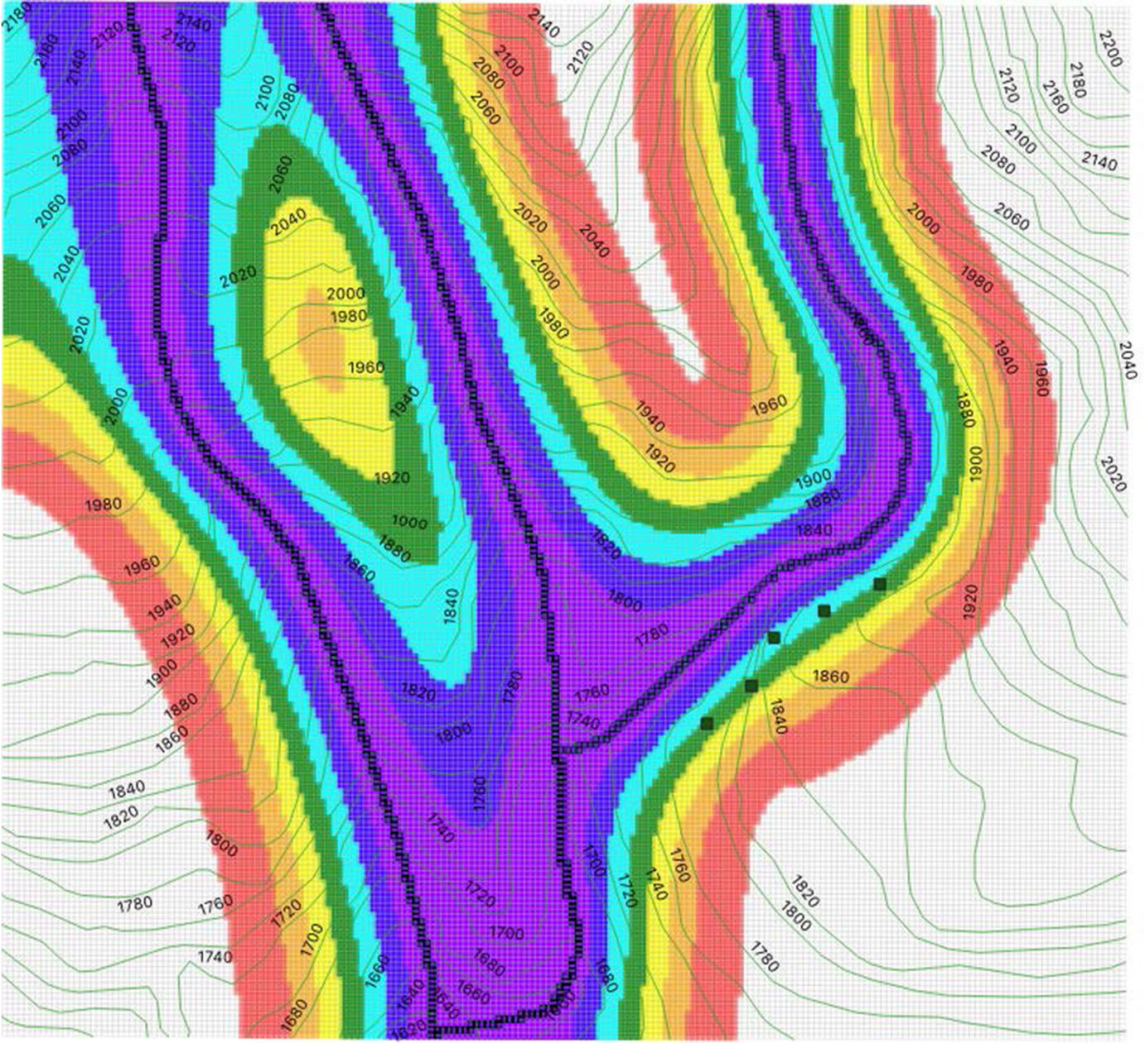
Heat map of the density of individuals. We use black for the river edges in order to make them distinguishable. We use the 7 colors of the rainbow: the most energetic color (violet) represents the higher density and the less energetic color (red) represents the lower density. The scale of colors is accompanied with a degradation of the intensity. Red  represents an expected number of individuals in the interval (0, *K*/7]; orange  represents an expected number of individuals in (*K*/7, 2*K*/7]; yellow  represents an expected number of individuals in (2*K*/7, 3*K*/7] ; green  represents an expected number of individuals in ( 3*K*/7, 4*K*/7 ] ; cyan  represents an expected number of individuals in (4*K*/7, 5*K*/7]; blue  represents an expected number of individuals in ( 5*K*/7, 6*K*/7]; violet  represents an expected number of individuals in (6*K*/7, *K*]. Recall the definition of *K* in (3.1) and the text below it (Color figure online)

In Ballesteros and Garro (2022) (Figs. 3, 4, 5 and 6), we show that the Gibbs sampling simulation leads us to an (approximate) minimizer in the free case (g=0), see Fig. 3 in Ballesteros and Garro (2022), which is a state that is essentially constant. Increasing the parameter *g* produces Gibbs sampling simulations that concentrate in the river to the point that when *g* is large enough states are supported in a very small vicinity of it (Figs. 4, 5 and 6 in Ballesteros and Garro (2022)). This shows that we have a global control of the measures that we use and we can increase *g* until we approximately match the year average of the total number of collected individuals on the parcels (which equals 0.3), and this value—we denote by $g_0$—is our initial estimation of *g*:

4.26 $$\begin{aligned} \text {Expected number of individuals on}\,P \approx \frac{1}{{\mathcal {M}}} \sum _{n=990001\times m_4}^{1000000\times m_4} \sum _{p \in P} \omega ^{i,n; g}_p \approx 0.3. \end{aligned}$$

The global control of the measures that we refer above is achieved only in the low temperature regime when the Gibbs sampling simulations produce low energy states. However, the role of the low temperature is not simple: a high value of the temperature implies that the states obtained from Gibbs sampling do not necessarily have low energies (the Gibbs measure is not highly concentrated on such states). A very small temperature is also problematic because Gibbs sampling generally gets stuck on states that maximize local conditional probabilities but not the global measure density (recall that minimal energies feature maximum probability), see Fig. 14 in Ballesteros and Garro (2022). The solution to this is called annealing (see Geman and Geman (1993); Hajek (1988)) which is a temperature descending scheme combined with Gibbs sampling. In Hajek (1988), it is proved that (theoretically) the temperature has to descend logarithmically starting from a huge number, in general. In our case this makes the requirements of Hajek (1988) impossible to achieve. However, numerical experiments using the clever choice of the cell ordering that we present in this section allow us to have a dramatic descent of the energy in the Gibbs sampling simulations (see Figs. 4 and 5). Our annealing scheme reduces to only one change of the temperature. We chose these two temperatures empirically with the help of many trial simulations. The fist selection of the temperature is fixed in order to satisfy (4.26). The state derived in (4.26) serves as an initial state for a new Gibbs sampling algorithm with a lower temperature that allows us to reduce even more the energy. We denote by $\varvec{T}_2$ this second choice of the temperature. With the new initial state in (4.26) and temperature $\varvec{T}_2$, we iterate $1000000\times m_4$ new Gibbs sampling simulations and use different values of *g* around $g_0$ in order to improve (4.26) with the very last ${\mathcal {M}}$ iterations. The value of *g* that gives the best estimates is denoted by ${\hat{ \varvec{g}}}$ and we set ${\hat{ \varvec{T}}}: = \varvec{T}_2$.

##### Remark 4.8

Figures 4 and 5 show an abrupt descent of the energy in the first iterations (they are the most important ones). A second abrupt descent of the energy is visible around the iteration 1000000, which is the point when the temperature changes to the value $\varvec{T}_2$. The message that we want to convey with these figures is the dramatic descent of the energy that the Gibbs sampling algorithm produces. The precise values of the energies are not relevant for our purposes. We prefer to not chose logarithmic scales because they make the message we want to communicate more difficult to visualize.

### Year-Average Densities per Cell and Graphical Representation

Once ${\hat{ \varvec{T}}}$ and ${\hat{ \varvec{g}}}$ are determined, we calculate $1000000\times m_4$ Gibbs sampling iterations with initial state zero, coupling constant ${\hat{ \varvec{g}}}$ and temperature $T_1$. We take the average of the last ${\mathcal {M}}$ states. We choose this average as the initial state for a new Gibbs sampling sequence with $1000000\times m_4$ iterations, using ${\hat{ \varvec{T}}}$ and ${\hat{ \varvec{g}}}$. Finally, we denote by

4.27 $$\begin{aligned} \varvec{\omega } \end{aligned}$$

the average of the last ${\mathcal {M}}$ states (from the $2000000\times m_4$ iterations).

The value ${\varvec{\omega }((i,j)) \equiv } \varvec{\omega }_{i,j}$ represents the expectation value of the year-average number of individuals on the cell (*i*, *j*). Since $\varvec{\omega }_{i,j}$ does not represent a realization of the number of individuals on the cell (*i*, *j*) (as in Ballesteros and Garro (2022)), we need to define more sophisticated graphical visualizations. In the next sections we explain this.

#### Individuals Depicted by Dots

In this section we introduce a graphical representation of the state $\varvec{\omega }$ defined in (4.27).

As we mention above, $\varvec{\omega }_{i,j}$ is an estimation of the expectation value of the year-average number of individuals on the cell (*i*, *j*). We use this information to get graphical representations of the individuals that we might observe in the region.

Simulated Individuals Observed During the Whole Year First, we denote by (recall (4.27))

$$\begin{aligned} \varvec{\omega }_{Y}:= 10 \varvec{\omega }, \end{aligned}$$

where

4.28 $$\begin{aligned} (10 \varvec{\omega })_{i,j} = 10 \varvec{\omega }_{i,j}. \end{aligned}$$

Then, $( \varvec{\omega }_{Y} )_{i,j}$ represents an estimation of the expected value of individuals observed during the whole year (10 field trips, see the text above (3.1)) on the cell (*i*, *j*).

For every (*i*, *j*), we simulate a random number $\varvec{n}_{i,j} \in \{0,1, 2, 3, \dots \}$ according to a Poisson distribution with expected value $( \varvec{\omega }_{Y} )_{i,j}$. Then we define

$$\begin{aligned} \varvec{n}: = ( \varvec{n}_{i,j} )_{ \text {for all cells}\; (i,j) }, \end{aligned}$$

which is a state constructed randomly with values in $\{0,1, 2, 3, \dots \}$, and $\varvec{n}_{i,j}$ represents the number of individuals that could have been seen on the cell (*i*, *j*), in the 10 trips described above Equation (3.1). In Sect. 5.3, we report our result graphically depicting $\varvec{n}_{i,j}$ dots on the cell (*i*, *j*), for every *i*, *j*. The dots describe a realization of individuals that could have been seen during the whole 10 dates described above (3.1).

Simulated Year-Average Observations

For every (*i*, *j*), we simulate a random number $x \in [0,1]$ (uniformly distributed). If $x \le \varvec{\omega }_{i,j}$, then we choose $\varvec{m}_{i,j} =1$, otherwise $\varvec{m}_{i,j} =0$. Then we define

$$\begin{aligned} \varvec{m}: = ( \varvec{m}_{i,j} )_{ \text { for all cells}\; (i,j) }, \end{aligned}$$

which is a state constructed randomly with values in $\{0,1\}$, it represents a realization of possible observations in the full region in a date randomly chosen from the 10 dates where the fieldwork was carried out (see the text above Equation (3.1)). In Sect. 5.3, we report our result graphically depicting $\varvec{m}_{i,j}$ dots on the cell *i*, *j*, for every *i*, *j*. The dots describe a realization of individuals that in average over the year could have been seen in one field trip.

#### Heat Maps

We use a heat map to depict the values $\varvec{\omega }_{i,j}$ of $\varvec{\omega }$ (recall (4.27)). We utilize the 7 colors of the rainbow: red, orange, yellow, green, cyan, blue and violet (this order corresponds to an ascending energy of the colors). Every cell (*i*, *j*) is colored with one of these colors depending on the values of $\varvec{\omega }_{i,j}$ in such a way that the color is constant on intervals of the form $\varvec{\omega }_{i,j} \in ( \frac{s}{7}K, \frac{s+1}{7}K ],$ $s \in \{0, \cdots , 6 \},$ and the energy of the color increases as $\varvec{\omega }_{i,j}$ increases. We, additionally, increase the intensity of the color as the energy increases.

## Results

### Temperature and Iterations (Free Case)

From the procedure described in Sect. 4.6, we obtain that

5.1 $$\begin{aligned} T_1 = 0.00000475\qquad \text {and}\qquad {\hat{ \varvec{T}}}=0.00000002. \end{aligned}$$

In Fig. 4 we present the graphic of the free energies of 2000000 Gibbs sampling iterations, in every region (as described in Sect. 4.6). It can be clearly seen that the energies descend dramatically and stabilize near the minimum energy (zero).

### The Coupling Constant ${\hat{ \varvec{g}}}$

We use the method described in Sect. 4.6.2 in order to obtain that

5.2 $$\begin{aligned} {\hat{ \varvec{g}}} := 0.000000325. \end{aligned}$$

In Fig. 5, we present the graphic of the energies of 2000000 Gibbs sampling iterations in every region (as described in Sect. 4.6.2). It can be clearly seen that the energies descend dramatically and stabilize near zero.

### Main Results: Distribution and Abundance of Individuals

**Fig. 7:**
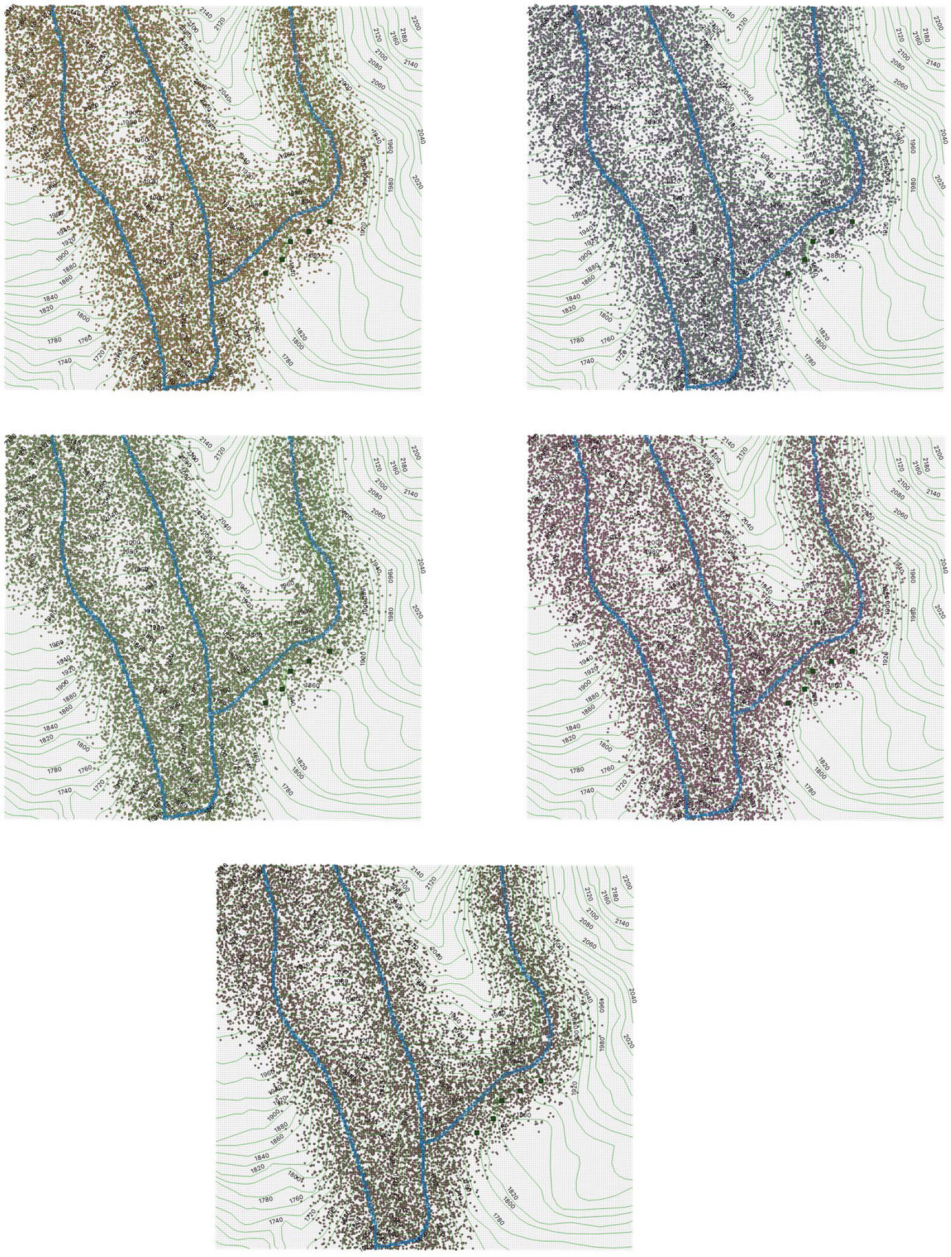
Individuals that could be seen during the 10 dates (5 realizations of $\varvec{n}$)

**Fig. 8:**
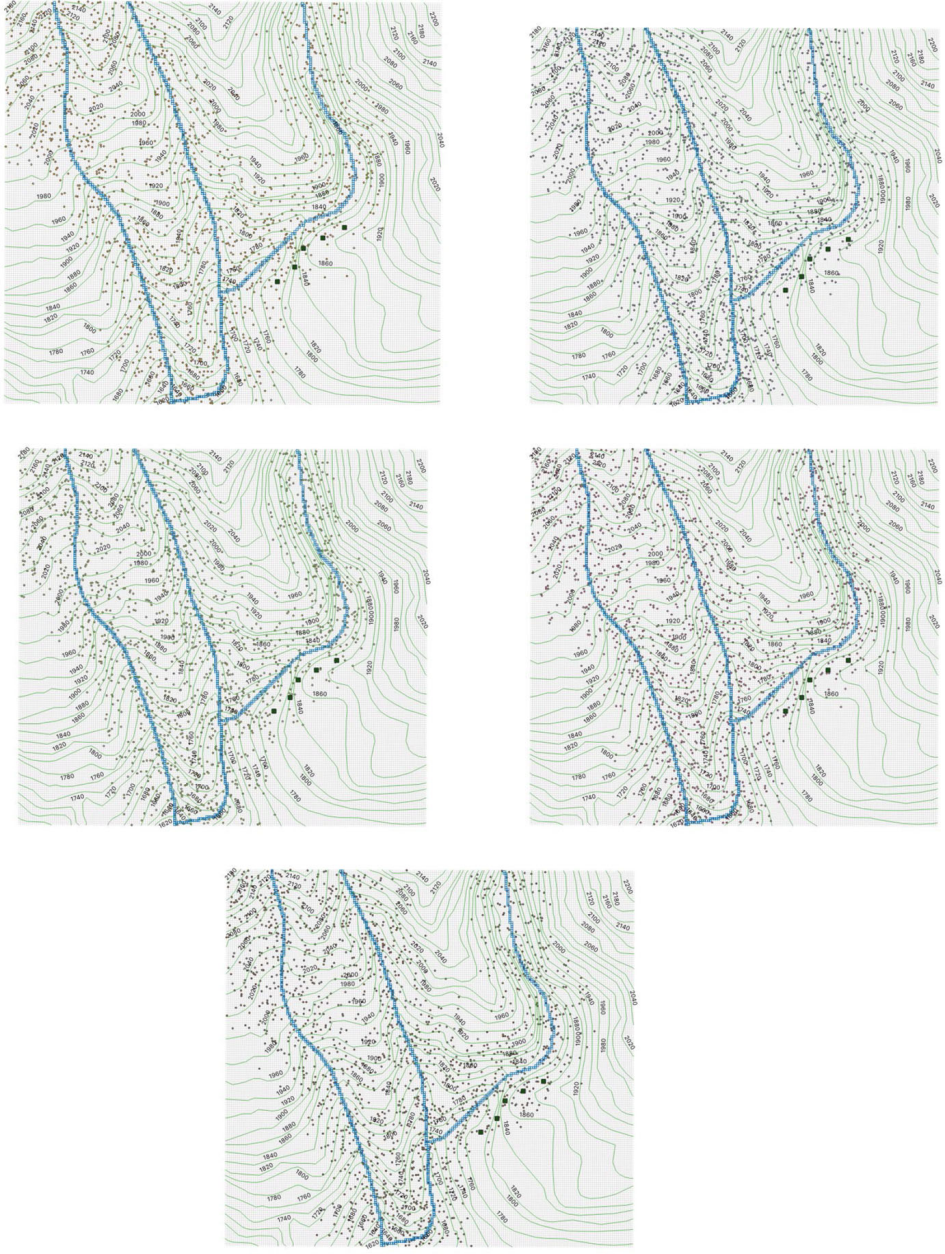
Individuals that could be seen in one field trip in the 10 dates (5 realizations of $\varvec{m}$)

Our main results are the graphical representations of the simulations that we obtain, describing the distribution and abundance of individuals on the region, as explained in Sects. 4.7.1 and 4.7.2:

- In Fig. 7, we represent with dots each individual that could be seen during the 10 dates reported above (3.1). We present 5 figures according to 5 realizations of $\varvec{n}$.
- In Fig. 8, we plot realizations of possible observations in the full region in a date randomly chosen from the 10 dates where the fieldwork was carried out. We present 5 figures according to 5 realizations of $\varvec{m}$.
- In Fig. 6, we provide a heat map as explained in Sect. 4.7.2. The density of individuals is higher on the river, and it decreases as the distance to the river increases. The species *Plectrohyla Guatemalensis* is attracted to the river, but it is not strongly attracted. This is different from other species of the genus Plectrohyla, such as Plectrohyla Sagorum which can only be found in a small neighborhood of the river.

## Conclusions

We estimate the distribution and abundance of the species *Plectrohyla Guatemalensis* in a region located in the nature reserve Tacana Volcano. It is near threatened and there is little known information about it. Our work contributes to the knowledge of this species and we hope that it might help its preservation. We obtain that individuals are attracted to the river, but the attraction force is much weaker than other species with the same genus such as Plectrohyla Sagorum. From the mathematical side, we present a method that can be used to describe from near threatened species to critically endangered species (based on the model introduced in Ballesteros and Garro (2022)). This is a hard problem, because the extremely low density of individuals that occurs in such situations makes it very difficult to reconstruct a probability distribution. In our case, there are in average no more than 2 individuals collected every field trip (and standard methods frequently use data from only one field sampling). It is important to remark that the region that we study is a canyon with a very complicated orography and, therefore, the field work is very difficult. Although we carried out an intensive and prolonged fieldwork (10 trips in one year), the data that we were able to obtain outside the river was extremely reduced. The neighborhood of the river is difficult to access because there are rock walls and a heavy vegetation. Moreover, it is nearly impossible to observe individuals far away from the river due to the low density. Taking onto account these restrictions, we decided to consider only one parameter to be estimated (namely, the coupling constant *g* that measures the strength of the attraction force). Inspired by the harmonic oscillator in quantum mechanics, we chose a quadratic potential well. This is usual in physics in many situations because the quadratic term is the leading order term in the Taylor series around a minimum. Since the quadratic behavior is fixed, only data at certain distances from the river is required for the estimation of *g*. And this is the only data available because in the neighborhood of the river there is only one walking path surrounded steep hillsides and rock walls. Despite complicated orography, our study region has important advantages. One of them is that in this region we were able to find endangered species of amphibians (this is very difficult to achieve) and another one is that the orography itself modulates the density of individuals and this is clearly observed in Fig. 6. In future work, we will study species that are not endangered in other regions of Mexico in such a way that we might be able to collect enough information in order to consider more complicated potential wells (we can even consider neural networks). We finally remark that, as we already mentioned in our previous work Ballesteros and Garro (2022), our method controls global probability measures in the low temperature regime. This is a difficult task because Gibbs sampling generally gets stuck on states that maximize local conditional probabilities but not the global measure density (recall that minimal energies feature maximum probability). Such states are abundant, in Figure 14 in Ballesteros and Garro (2022) we give an example of them.
